# Molecular docking analysis of beta-catenin with compounds derived from Lycopersicon esculentum 

**DOI:** 10.6026/97320630016801

**Published:** 2020-11-30

**Authors:** Jayaraman Selvaraj, Veeraraghavan Vishnupriya, Hussain Sardar, Janardhana Papayya Balakrishna, Josephine Rex, Surapaneni Krishna Mohan, Periyasamy Vijayalakshmi, Rajagopal Ponnulakshmi

**Affiliations:** 1 Department of Biochemistry, Saveetha Dental College and Hospitals, Saveetha Institute of Medical and Technical Sciences, Saveetha University, Chennai - 600 077, India; 2 Department of Biotechnology, Government Science College, Chitradurga-577501, Karnataka, India; 3 Department of Stem Cell Biology, Stellixir Biotech Pvt Ltd, No.V-31, 2nd floor, 10th Main Road, Peenya 2nd Stage Industrial Area, Bangalore - 560058, Karnataka, India; 4 Department of Biochemistry, Panimalar Medical College Hospital & Research Institute, Varadharajapuram, Poonamallee, Chennai - 600 123, India; 5 Department of Biochemistry and Department of Clinical Skills & Simulation, Panimalar Medical College Hospital & Research Institute, Varadharajapuram, Poonamallee, Chennai - 600 123, India; 6 PG & Research Department of Biotechnology & Bioinformatics, Holy Cross College (Autonomous), Trichy - 620002, Tamil Nadu, India; 7 Central Research Laboratory, Meenakshi Academy of Higher Education and Research (Deemed to be University), Chennai - 600 078, India

**Keywords:** Colon Cancer, Lycopersicon esculentum, Beta-catenin, Molecular docking

## Abstract

Beta-catenin is linked with colorectal cancer (CRC). Therefore, it is of interest to design and develop novel compounds to combat CRC. Hence, we document compounds (chlorogenic acid, gallic acid, protocatechuic acid, quercetin and vanillic acid) from Lycopersicon esculentum with optimal binding features for further consideration.

## Background

Colon and rectum carcinoma is a significant health concern globally, with more than 700,000 deaths each year [1 - see PDF]. Nearly 30% of CRC patients develop recurrences despite curative surgery [2]. Infections in various sites or remote lymph nodes (remote recurrences) have a serious impact on the CRC prognosis. Wnt signal pathways regulate cell proliferation, migration and destiny during embryonic development. It is known that Wnt signalling pathways, Wnt / b-catenin (canonical pathway) is mutated in nearly 90% of colorectal cancers (CRCs) [3]. The vast majority of colorectal cancers have mutations in Wnt pathway genes like adenomatous polyposis coli (APC) and β-catenin (CTNNB1) genes. The APC gene was previously identified as causative of family adeno matouspolyposis (FAP) syndrome [4,5] and often mutated in sporadic colo-rectal cancers. APC mutations occur similarly in replication error (RER) positive and negative colorectal cancers [6,7] and are the earliest genetic event in the so-called ade-noma-carcinoma sequence. Therefore, it is of interest to design and develop novel compounds to combat CRC.

## Materials and Methods:

### Preparation of Receptor for docking:

The three-dimensional structure of Beta-catenin from Homo sapiens PDB: ID 1JDH downloaded from the protein databank (PDB) was used in this study [8].

### Preparation of Ligands:

10 known compounds from the tomato plant have been selected from the literature for this Study (Table 1 - see PDF). The structures of these compounds were obtained from the PubChem Compound Database in the Spatial Data File (.SDF) format and converted to the PDB file format using the Online Smile Translator. Energy minimization of ligands was completed with ChemBio 3D Ultra 12.0 using standard procedure.

### Molecular Docking of ligands with Beta-catenin:

Molecular docking analysis of beta-catenin with compounds derived from Lycopersicon esculentum was completed using the Hex 8.0.0. Docking software [9].

## Results and Discussion:

**Figure 1:**
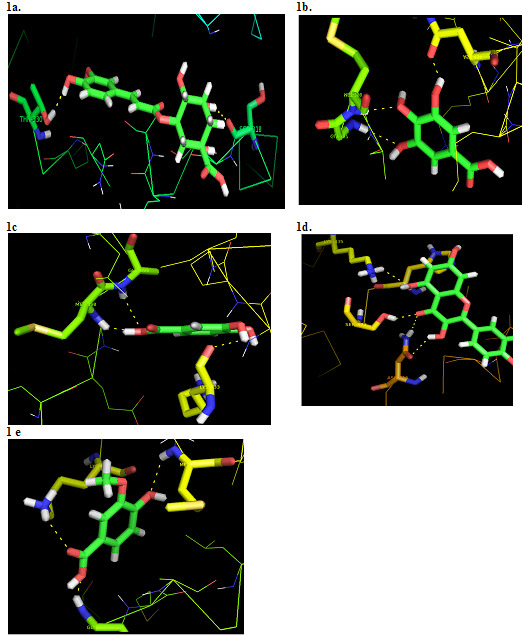
Molecular Interaction of β-catenin with (a) Chlorogenicacid b) Gallic acid, (c) Protocatechuic acid; (d) Quercetin and (e) Vanillic acid is shown

The HEX docking results of selected compounds with beta catenin showed that they would have a great docking score (E-total value) as shown in Table 2 (see PDF). The more negative the value, the more stable the complex is and the more binding affinity. According to energy funnel theory, less energy represents extremely reliable conformation. More negative Etotal value indicates that there must be a substantial relationship between ligand and receptor, leading to the activation of receptor activity [10]. The molecular docking findings of the selected five compounds (chlorogenic acid, gallic acid, protocatechuic acid, quercetin & vanillic acid) with beta-catenin were shown in Figure 1. We selected the best five of the 12 compounds. All the five compounds have good docking score and E-total value. The molecules that bind to the receptor inhibit its function for further consideration. HEX 8.0 has shown that the selected five compounds have a binding value of -170.22 to -255.21 kJ/mol. Molecular docking data shows that these five compounds are potent beta-catenin inhibitor.

The resultant docked complexes were further examined with pymol to show the H-bond interactions between the selected compounds and the beta-catenin protein. The findings showed that many of the compounds displayed significant H-bonding interactions with beta-catenin through the amino acids SER-318, THR-330, GLY-397, MET-398, ASN-434, GLU-399, LYS-433, LYS-435, SER-473, ASN-516 and MET-437 (Figure 1). Higher binding molecular docking scores and strong molecular interactions recommended that such compounds are effective candidate for inhibition of beta catenin for further consideration.

## Conclusion

We document compounds (chlorogenic acid, gallic acid, protocatechuic acid, quercetin and vanillic acid) from Lycopersicon esculentum with optimal binding features for further consideration to combat CRC.
